# What Factors Are Most Closely Associated With Mood Disorders in Adolescents During the COVID-19 Pandemic? A Cross-Sectional Study Based on 1,771 Adolescents in Shandong Province, China

**DOI:** 10.3389/fpsyt.2021.728278

**Published:** 2021-09-16

**Authors:** Ziyuan Ren, Yaodong Xin, Zhonglin Wang, Dexiang Liu, Roger C. M. Ho, Cyrus S. H. Ho

**Affiliations:** ^1^Department of Medical Psychology and Ethics, School of Basic Medicine Sciences, Cheeloo College of Medicine, Shandong University, Jinan, China; ^2^School of Statistics and Management Shanghai University of Finance and Economics, Shanghai, China; ^3^School of Physical Science, University of California, Irvine, Irvine, CA, United States; ^4^Department of Psychological Medicine, Yong Loo Lin School of Medicine, National University of Singapore, Singapore, Singapore; ^5^Institute of Health Innovation and Technology (iHealthtech), National University of Singapore, Singapore, Singapore

**Keywords:** COVID-19, mood disorders, sleep quality, resilience, adolescents, GBDT, SHAP value

## Abstract

**Background and Aims:** COVID-19 has been proven to harm adolescents' mental health, and several psychological influence factors have been proposed. However, the importance of these factors in the development of mood disorders in adolescents during the pandemic still eludes researchers, and practical strategies for mental health education are limited.

**Methods:** We constructed a sample of 1,771 adolescents from three junior high middle schools, three senior high middle schools, and three independent universities in Shandong province, China. The sample stratification was set as 5:4:3 for adolescent aged from 12 – 15, 15 – 18, 18 – 19. We examined the subjects' anxiety, depression, psychological resilience, perceived social support, coping strategies, subjective social/school status, screen time, and sleep quality with suitable psychological scales. We chose four widely used classification models-k-nearest neighbors, logistic regression, gradient-boosted decision tree (GBDT), and a combination of the GBDT and LR (GBDT + LR)-to construct machine learning models, and we utilized the Shapley additive explanations value (SHAP) to measure how the features affected the dependent variables. The area under the curve (AUC) of the receiver operating characteristic (ROC) curves was used to evaluate the performance of the models.

**Results:** The current rates of occurrence of symptoms of anxiety and depression were 28.3 and 30.8% among the participants. The descriptive and univariate analyses showed that all of the factors included were statistically related to mood disorders. Among the four machine learning algorithms, the GBDT+LR algorithm achieved the best performance for anxiety and depression with average AUC values of 0.819 and 0.857. We found that the poor sleep quality was the most significant risk factor for mood disorders among Chinese adolescents. In addition, according to the feature importance (SHAP) of the psychological factors, we proposed a five-step mental health education strategy to be used during the COVID-19 pandemic (sleep quality-resilience-coping strategy-social support-perceived social status).

**Conclusion:** In this study, we performed a cross-sectional investigation to examine the psychological impact of COVID-19 on adolescents. We applied machine learning algorithms to quantify the importance of each factor. In addition, we proposed a five-step mental health education strategy for school psychologists.

## Introduction

Since it was first reported by the World Health Organization (WHO) on December 31, 2019, the coronavirus disease 2019 (COVID-19) has cumulatively infected more than 130 million people, and it caused nearly 3 million deaths worldwide as of April 14, 2021 (1). Extensive research has shown that major adverse social events, such as terrorist attacks (2, 3), economic crises (4, 5), natural disasters (6, 7), and pandemics (8–10), can have a severe impact on people's mental health. As the most severe pandemic in recent decades, COVID-19 rapidly spread across the world, and the social impacts have far exceeded those of other pandemics, including the severe acute respiratory syndrome (SARS), Middle East respiratory syndrome (MERS), and novel influenza A (H1N1) pandemics. Therefore, the issue of mental health during the COVID-19 pandemic has received considerable critical attention.

Adolescents are members of society that are vulnerable to the psychological impacts of COVID-19. Many countries have closed schools to prevent the spread of the disease, resulting in multiple consequences for adolescents' daily lives: increased screen time, worry for their families and countries, social distancing, and home confinement. Given that adolescence is a critical neurobiological period in the development of higher-order cognition, stressful experiences, such as lockdowns and social isolation, can lead to the early development of multiple psychiatric disorders (11). Hawke et al. performed a retrospective study that compared adolescents' mental health before and after the onset of the COVID-19 pandemic in Canada and found that the symptoms of depression and anxiety significantly increased (12). In China, 43.7, 37.4, and 31.3% of high school students showed symptoms of anxiety, depression, or a combination of depression and anxiety during the COVID-19 lockdowns (13).

COVID-19 cases recently rose for a seventh consecutive week with a rate of over 4.5 million new cases per week (1). According to a predictive model based on the Italian population, even if a combination of fast vaccination rollouts and restrictive non-pharmaceutical interventions is implemented, there will still be an estimated 18,000 deaths from April 2021 to January 2022 (14). Prolonged or intermittent lockdowns are predicted to last until 2022 (15). There is an urgent need to recognize the mechanisms that potentially influence adolescents' mental health. Many psychosocial factors, such as psychological resilience and social support, have been demonstrated as crucial regulators in multisystemic processes that can contribute to positive outcomes (16, 17). In addition, demographic characteristics, sleep quality, and screen time were reported to influence a wide range of cognitive and emotional functions (18–22). However, the importance of these factors in the development of mood disorders in adolescents during the pandemic still eludes researchers, and practical strategies for mental health education are limited.

To this end, we performed a cross-sectional investigation based on 1,771 adolescents from six independent middle schools and three independent universities in Shandong province to examine the psychological impacts of COVID-19 on adolescents. We focused on psychological resilience, perceived social support, coping strategies, subjective social/school status, screen time, and sleep quality, constructed machine learning models for these factors, and applied the SHAP (Shapley additive explanations value) to quantify the impact of each factor. In addition, we proposed a five-step mental health education strategy for school psychologists.

## Materials and Methods

### Ethics Statement

The Ethics Committee of Shandong University approved this human-involved survey (ECSBMSSDU2021-1-067). Throughout the whole study, we strictly followed the Declaration of Helsinki, did not collect or involve any subject-identifying information, and electronically obtained informed consent from all of the participants.

### Data Collection

We performed this cross-sectional study to examine the mental status of Chinese adolescents during the COVID-19 pandemic and to explore the potential mechanisms of the impact of COVID-19 on adolescents' mental health. We collected data between 20 April and 30 April, 2021 by using an online questionnaire website (www.wenjuan.com). According to a statement from the WHO, individuals aged 10 – 19 years were defined as adolescents (23). According to the ethics requirements of Shandong University, children-involved psychological investigation is strictly limited, so we did not include children in this study, which is one of our limitations. The adolescents who were aged between 12 and 19 years old were invited. We hypothesized that younger adolescents were more susceptible to mood disorders. Therefore, the designed sample stratification was set as 5:4:3 for adolescent from junior high school, senior high school, and university. We recruited participants from three junior high schools (predominantly aged between 12 and 15 years old), three senior high schools (predominantly aged between 15 and 18 years old), and three colleges (predominantly freshmen of 18 – 19 years old) (considering that the academic level of the school might influence students' mental status, we selected schools of different levels on a 1:1:1 scale). The details of the included schools are provided in Supplemental Table 1. In compliance with the requirements of the Ethics Committee of Shandong University, adolescents who have recently suffered severe negative life events or were diagnosed with severe psychological disorders were not to be recruited in the study as subjects. In this study, we defined “effective questionnaire” as the retrieved questionnaires which had been completed without blanks and having no obvious or doubtful responses. For example, if a subject chose the first option in all questions, this questionnaire was defined as ineffective as this type of consistent responses across all questions are considered rare and doubtful. We in all invited 1,776 subjects to participate in this investigation, among which 260/1,776 subjects completed the paper questionnaires and 1,511/1,776 subjects completed the online questionnaires. All of the paper questionnaires were effective. Two online received responses left blanks and three cast obvious mistakes. Therefore, we collected 1,771 effective questionnaires in total. The actual sample stratification was around 5:4:3 (865:511:395) for adolescents from junior high school, senior high school, and university.

## Designed Questionnaire

### Basic Personal Characteristics

We collected the participants' demographic characteristics, including their gender (male/female) and grade (from grade 7 to grade 12, plus the freshman year in college). We investigated the impact of COVID-19 on the participants' families, including having relatives or friends who had been infected (yes/no) and having relatives or friends who had been quarantined (yes/no).

We also investigated two aspects of personal living styles: physical activity and screen time. Physical activity was assessed according to the average time spent participating in moderate to vigorous physical activity over the last week. The amount of physical activity was categorized into four levels: <= 30, 30 – 60, 60 – 120, and more than 120 min. According to the WHO, physical activity includes play, games, sports, transportation, chores, recreation, physical education, or planned exercise in the context of family, school, and community activities for children and young people (24). Screen time was assessed according to the average time per day spent on electronic devices (including televisions, smartphones, tablets, and computers), and this was categorized into four levels: <= 30, 30 – 60, 60 – 120, and more than 120 min. During this investigation, the subjects from six middle schools and three universities were all studying at school but not online. Therefore, they did not have to use electric devices to attend classes. Besides, if they utilized computers or smart phones to study in their rest time, this time was included in the screen time.

### Assessment of Anxiety and Depression

We utilized the General Anxiety Disorder-7 (GAD-7, Chinese version) to assess the symptoms of anxiety experienced in the previous 2 weeks. The GAD-7 is a self-reported and simple anxiety measurement with high reliability and validity (25), and its Cronbach's α coefficient in Chinese adolescents was previously found to be 0.917 (26). The GAD-7 includes seven four-point Likert scales (ranging from “not at all” [0 points] to “nearly every day” [three points]) that describe the typical symptoms of GAD. Individuals with scores of 5 – 9, 10 – 14, 15 – 21 showed mild, moderate, or severe anxiety symptoms, respectively. In the current study, the Cronbach's α coefficient was 0.916.

We utilized the Patient Health Questionnaire-9 (PHQ-9, Chinese version) to assess the depressive symptoms experienced in the previous 2 weeks. The PHQ-9 is a self-reported depression measurement with high reliability and validity (27), and its Cronbach's α coefficient in Chinese adolescents was previously found to be 0.869 (26). The PHQ-9 includes nine four-point Likert scales (ranging from “never” [0 points] to “almost every day” [three points]) that describe the typical symptoms of depression. Individuals with scores of 5 – 9, 10 – 14, 15 – 19, or 20 – 27 showed mild, moderate, moderately severe, or severe depressive symptoms, respectively. In the current study, the Cronbach's α coefficient was 0.918.

### Assessment of Sleep Quality

We utilized the Self-Rating Scale of Sleep (SRSS) to assess sleep quality. The SRSS is a self-reported sleep quality measurement tailored for Chinese people (28). Its reliability and validity have been proven by several previous studies (29, 30). The SRSS includes ten items with a score ranging from the best sleep quality (one point) to the worst sleep quality (five points). Individuals with total scores of 23 – 29, 30 – 39, or 40 – 50 showed mild, moderate, or severe sleep disturbance, respectively. In the current study, Cronbach's α was 0.784.

### Assessment of Subjective Social Status

We utilized the Subjective Social Status-Adolescent (SSS-A, Chinese version) scale, which was derived from the MacArthur Subjective Social Status scale (31, 32). The SSS-A was developed explicitly for measuring adolescent's SSS with a high reliability and validity. The SSS-A includes two ten-point Likert scales ranging from “the lowest” (one point) to “the highest” (10 points) to evaluate the participants' subjective social status and campus status, respectively.

### Assessment of Psychological Resilience

We utilized the Connor–Davidson Resilience Scale-10 (CD-RISC-10, Chinese version) to assess the participants' psychological resilience over the last month. The CD-RISC-10 is a self-reported psychological resilience measurement that includes ten five-point Likert scales that range from “not true at all” (0 points) to “true nearly all the time” (four points) (33). The higher the total score, the higher the psychological resilience. Many previous studies have demonstrated its reliability (Cronbach's α > 0.9) and validity (34, 35). In this study, the Cronbach's α was 0.950.

### Assessment of Coping Strategies

We utilized a recently developed eight-item coping strategy measurement derived from the original Ways of Coping (36, 37). and tailored for Chinese adolescents. This measurement includes eight four-point Likert scales that range from “never used” (one point) to “used a great deal” (four points), including five adaptive coping strategies and three maladaptive coping strategies. The Cronbach's α was 0.78. The higher the total score, the better the coping strategy. In this study, the Cronbach's α was 0.862.

### Assessment of Perceived Social Support

We utilized the Multidimensional Scale of Perceived Social Support (MSPSS, Chinese version) to investigate the social support of each participant; this is a self-reported and highly effective social support measurement (38). The reliability and validity of the MSPSS were proven in a population of Chinese adolescents (22, 39, 40). The MSPSS includes twelve seven-point Likert scales that range from “very strongly disagree” (one point) to “very strongly agree” (seven points), which could be classified into three subscales to evaluate the perceived social support from the family, friends, and a significant other, respectively. The higher the total score, the higher the perceived social support. Xu et al. reported that the Cronbach's α of the total scale and three subscales were 0.958, 0.905, 0.915, and 0.934, respectively. In the current study, the Cronbach's α was 0.966.

## Statistics

### Descriptive and Univariate Analysis

First of all, it is necessary to use univariate analysis methods in order to explore the basic relations between features and labels. To be specific, we applied the Chi-square test for the binary and polytomous unordered features. In particular, for features that had cells with a count of, <5, we used Fisher's exact test to assess the accuracy. For polytomous ordered features, we calculated Somer's d coefficients in order to measure the consistency (that is, whether the two variables tend to move in the same or opposite directions). On the other hand, in terms of consistent features, we applied the Mann-Whitney *U* test to determine whether the average value of each feature was different between two labels. According to the results of the univariate analysis, we preprocessed the dataset to improve the models' performance.

### Machine Learning Models

It should be noted that this is a typical binary classification problem. Therefore, we chose four widely used classification models: *k*-nearest neighbors (KNN), logistic regression (LR), gradient-boosted decision tree (GBDT), and a combination of the GBDT and LR (GBDT+LR).

KNN: KNN is a widely utilized machine learning algorithm in the field of classification and regression prediction due to its simple implementation and outstanding performance (41). The KNN method stores all available instances and classifies new instances based on a similarity measure (such as distance functions) (42).

GBDT: GBDT is a new algorithm that combines decision trees and holistic learning techniques. Its basic idea is to combine a series of weak base classifiers into a strong base classifier (43, 44). In the learning process, a new regression tree is constructed by fitting residuals to reduce the loss function until the residuals are less than a certain threshold, or the number of regression trees reaches a certain threshold. The advantages of GBDT are good training effect, less overfitting, and flexible handling of various data types, including continuous and discrete values (45).

LR: LR is a supervised machine learning algorithm for learning classification and regression problems. It is one of the most widely used methods in health sciences research, especially in epidemiology (46). Many previous studies have shown that LR is effective in analyzing risk/protective factors in the field of psychological research (47–49).

GBDT+LR: GBDT+LR is a very novel machine learning algorithm for classification and regression problems. The performance of LR model depends strictly on feature extraction since LR can only find a linear relationship. Under this circumstance, using GBDT to firstly process the variables by finding their locations in trees' leaf nodes and encoding them into new features contribute to solving this problem. It helps to combine the information of the features automatically and improve the performance of the following LR model (50).

### Parameter Selection

To determine the optimal parameters, we used the sklearnGridSearchCV module to perform a grid search, the details of which are listed in the following. Firstly, we set up the optional ranges of all parameters. Secondly, we used five-fold cross-validation to test the AUC (area under the curve) on the validation dataset with the parameters in every possible combination. The combination that had the highest AUC value was considered the best. Five-fold cross-validation involved randomly dividing all of the datasets into five equal subsets and using four of them as the training dataset to fit the model. The effect of the model was measured with the AUC value, which we obtained by applying the model on the one subset that was left as a validation dataset.

### Model Evaluation

The AUC of the receiver operating characteristic (ROC) curves, sensitivity, and specificity (sensitivity = TP/ (TP + FN), specificity = TN/ (TN + FP), where TP represents true positive assignments, TN represents true negative assignments, FP represents false positive assignments, and FN represents false negative assignments) were used to evaluate the machine learning models.

To evaluate the effects of the machine learning models, we randomly split the dataset more than 100 times, with 85% as the training set and 15% as the test set. After splitting the dataset, we applied the four models mentioned above to fit the training set. By repeating the random splitting of the dataset, we countered the randomicity of the results. Then, we calculated the sample mean $\bar{X}$ and sample standard deviation *S* of the AUC value, sensitivity, and specificity of these models for the test set. The formulas listed below estimate the mean and the asymptotic 95% confidence intervals (95% CI) of these indices.


$$\begin{array}{l} \;\mu =\bar{X}\\ 95\%CI=\left(\bar{X}-1.96*S,\bar{X}+1.96*S\right) \end{array}$$

### Software

Our datasets were collected and descriptively analyzed using Excel 2016. Univariate analysis was performed using IBM SPSS Statistics 25. The machine learning models were constructed using the scikit-learn 0.24.2 package. A *P-*value of < 0.05 was considered statistically significant.

## Results

### The Rate of Occurrence of Anxiety and Depression Symptoms

The GAD-7 and PHQ-9 were utilized to evaluate the anxiety and depression among 1,771 participants. The rate of occurrence of anxiety symptoms was 28.3% (502/1,771). A total of 21.7% (385/1,771) of the participants showed mild anxiety symptoms, 4.5% (79 / 1,771) of the participants showed moderate anxiety symptoms, and 2.1% (38/1,771) of the participants showed severe anxiety symptoms. The rate of occurrence of depression symptoms was 30.8% (545/1,771). A total of 21.2% (375/1,771) of the participants showed mild depression symptoms, 5.6% (100/1,771) of the participants showed moderate depression symptoms, 2.7% (47/1,771) of the participants showed moderate to severe depression symptoms, and 1.3% (23/1,771) showed severe depression symptoms. In addition, 20.1% (356/1,771) of the participants showed both anxiety and depression symptoms. Then, we divided the 1,771 participants into two groups according to whether they were anxious or depressed for further factor analysis.

### Descriptive and Univariate Analyses of Demographic Characteristics, Personal Living Styles, and Psychological Factors

The results of the descriptive analysis of the 1,771 participants are presented in Table 1. As for the demographic characteristics, we found that adolescents who were female, were in graduating grades, and had relatives/friends that had infected by/quarantined with COVID-19 were more likely to experience anxiety/depression (*P* < 0.05). As for personal living styles, more physical activity and less screen time could prevent adolescents from experiencing anxiety/depression (*P* < 0.05). Among the included psychological factors, sleep quality, resilience, subjective social/school status, perceived social support (from family/friends/significant others), and adaptive coping strategies were protective factors, and maladaptive coping strategies were a risk factor (*P* < 0.05). The results of the descriptive analysis showed that all of the factors were statistically associated with mood disorders in the adolescents, which was rational and consistent with previous research. However, as we previously speculated, simple descriptive statistical methods cannot quantify the impact of each factor on anxiety/depression in adolescents, which limited the clinical significance of these factors. Therefore, we introduced machine learning methods in order to further analyze the importance of each factor.

**Table 1:** Descriptive analysis of the 1,771 samples.

**Variable (*n* = 1771)**	**All Participants (%)**	**Anxiety**		***P-value***	**Depression**		***P-value***
		**NO (%)**	**YES (%)**		**NO (%)**	**YES (%)**	
**Gender**							
Male	917 (51.8)	686 (74.8)	231 (25.2)	0.002	658 (71.8)	259 (28.2)	0.017
Female	854 (48.2)	583(68.3)	271 (31.7)	C	568 (66.5)	286 (33.5)	C
**Grade**							
7^th^ in middle school	196 (11.1)	145 (74.0)	51 (26.0)	0.000	146 (74.5)	50 (25.5)	0.000
8^th^ in middle school	596 (33.7)	428 (71.8)	168 (28.2)	C	417 (70.0)	179 (30.0)	C
9^th^ in middle school	73 (4.1)	37 (50.7)	36 (49.3)		46 (63.0)	27 (37.0)	
10^th^ in middle school	302 (17.1)	225 (74.5)	77 (25.5)		208 (68.9)	94 (31.1)	
11^th^ in middle school	78 (4.4)	58 (74.4)	20 (25.6)		54 (69.2)	24 (30.8)	
12^th^ in middle school	131 (7.4)	79 (60.3)	52 (39.7)		67 (51.2)	64 (48.9)	
Freshman in college	395 (22.3)	297 (75.2)	98 (24.8)		288 (72.9)	107 (27.1)	
**Having relatives or friends who have been infected**							
Yes	9 (0.5)	2 (22.2)	7 (77.8)	0.003	1 (11.1)	8 (88.9)	0.001
No	1,762 (99.5)	1,267 (71.9)	495 (28.1)	F	1,225 (69.5)	537 (30.5)	F
**Having relatives or friends who have been quarantined**							
Yes	116 (6.6)	63 (54.3)	53 (45.7)	0.000	64 (55.2)	52(44.8)	0.001
No	1,655 (93.5)	1,206 (72.9)	449 (27.1)	C	1,162 (70.2)	493(29.8)	C
**Physical activity**							
<30 min	547 (30.9)	354 (64.7)	193 (35.3)	0.000	332 (60.7)	215 (39.3)	0.000
30 – 60 min	816 (46.1)	611 (74.9)	205 (25.1)	S	606 (74.3)	210 (25.7)	S
60 – 120 min	251 (14.2)	181 (72.1)	70 (27.9)		179 (71.3)	72 (28.7)	
>120 min	157 (8.9)	123 (78.3)	34 (21.7)		109 (69.4)	48 (30.6)	
**Screen time**							
<30 min	570 (32.2)	427 (74.9)	143 (25.1)	0.023	408 (71.6)	162 (28.4)	0.014
30 – 60 min	392 (22.1)	281 (71.7)	111 (28.3)	S	282 (71.9)	110 (28.1)	S
60 – 120 min	265 (15.0)	186 (70.2)	79 (29.8)		182 (68.7)	83 (31.3)	
<120 min	544 (30.7)	375 (68.9)	169 (31.1)		354 (65.1)	190 (34.9)	
Sleep quality (mean [standard deviation])	18.75 (5.4)	17.27 (4.4)	22.51 (5.8)	0.000	16.9 (4.0)	22.92 (5.8)	0.000
				M			M
**Subjective social status (mean, [standard deviation])**							
Social status	5.8 (2.0)	5.92 (2.0)	5.51 (2.0)	0.000	5.98 (1.9)	5.41 (2.0)	0.000
				M			M
School status	6.18 (2.1)	6.4 (2.1)	5.62 (2.1)	0.000	6.49 (2.0)	5.46 (2.1)	0.000
				M			M
Psychological Resilience (mean (standard deviation))	25.57(9.1)	27.22(9.3)	21.4(7.1)	0.000	27.6(9.1)	21(7.1)	0.000
				M			M
**Perceived social support (mean [standard deviation])**							
Social support from the family	17.27 (5.3)	18.06 (5.2)	15.28 (5.1)	0.000	18.4 (5.0)	14.74 (5.2)	0.000
				M			M
Social support from the friends	16.83 (5.2)	17.56 (5.2)	14.98 (5.0)	0.000	17.84 (5.1)	14.56 (4.9)	0.000
				M			M
Social support from a significant other	16.73 (5.2)	17.53 (5.1)	14.71 (5.0)	0.000	17.82 (5.0)	14.28 (4.9)	0.000
				M			M
Total social support	50.83 (14.8)	53.16 (14.6)	44.96 (13.5)	0.000	54.06 (14.2)	43.58 (13.4)	0.000
				M			M
**Coping strategy (mean [standard deviation])**							
Adaptive coping strategy	15.56 (3.5)	16.12 (3.5)	14.13 (3.3)	0.000	16.24 (3.5)	14.02 (3.2)	0.000
				M			M
Maladaptive coping strategy	6.75 (2.4)	6.57 (2.4)	7.22 (2.3)	0.000	6.54 (2.5)	7.24 (2.3)	0.000
				M			M

*C, Chi-square test; F, Fisher's test; S, Somer's d test; M, Mann–Whitney test*.

### Data Preprocessing

As shown in Table 1, adolescents in the 7^th^, 8^th^, 10^th^, and 11^th^ grades, as well as those in the first year in college, had similar psychological statuses. The psychological conditions of adolescents in the 9^th^ and 12^th^ grades were significantly worse than those of other adolescents. It was not hard to find the reason: In China, 9^th^ and 12^th^ grade students are faced with the stress of gaining admission to higher education, i.e., senior high schools or colleges, thus causing relatively worse psychological statuses. Therefore, we converted the grade variable into the graduation grade variable. Adolescents who were in grades nine and 12 received a “Yes”, and those who were not received a “No”. For the two ordered variables, physical activity and screen time, and the other continuous variables, we found that the psychological conditions of students had approximately monotonic trends as these variables increased. Thus, no extra processes were performed for these features. In addition, the value of the total social variable was actually the sum of the values of the three variables above, which apparently indicated the existence of collinearity. Thus, we deleted this variable (total social support) and did not take its influence into account.

### Machine Learning Model Construction and Evaluation

**Figure 1:**
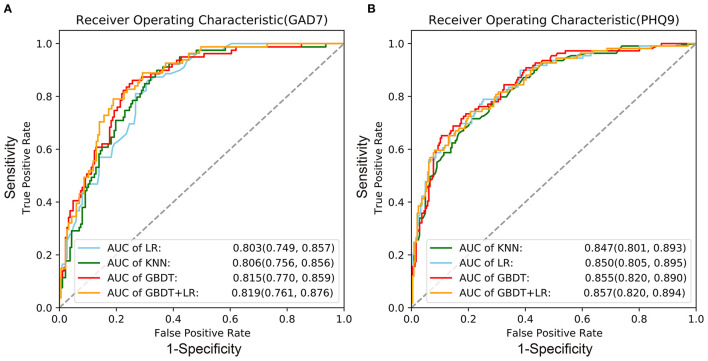
Receiver operating characteristic curves of the four machine learning models. **(A)** Anxiety (GAD-7); **(B)** depression (PHQ-9). KNN (k-nearest neighbors); LR (logistic regression); GBDT (gradient-boosted decision tree); GBDT+LR (a combination of GBDT and LR); AUC, area under the curve. 95%CI = (mean−1.96^*^ standard deviation, mean+1.96^*^ standard deviation).

**Figure 2:**
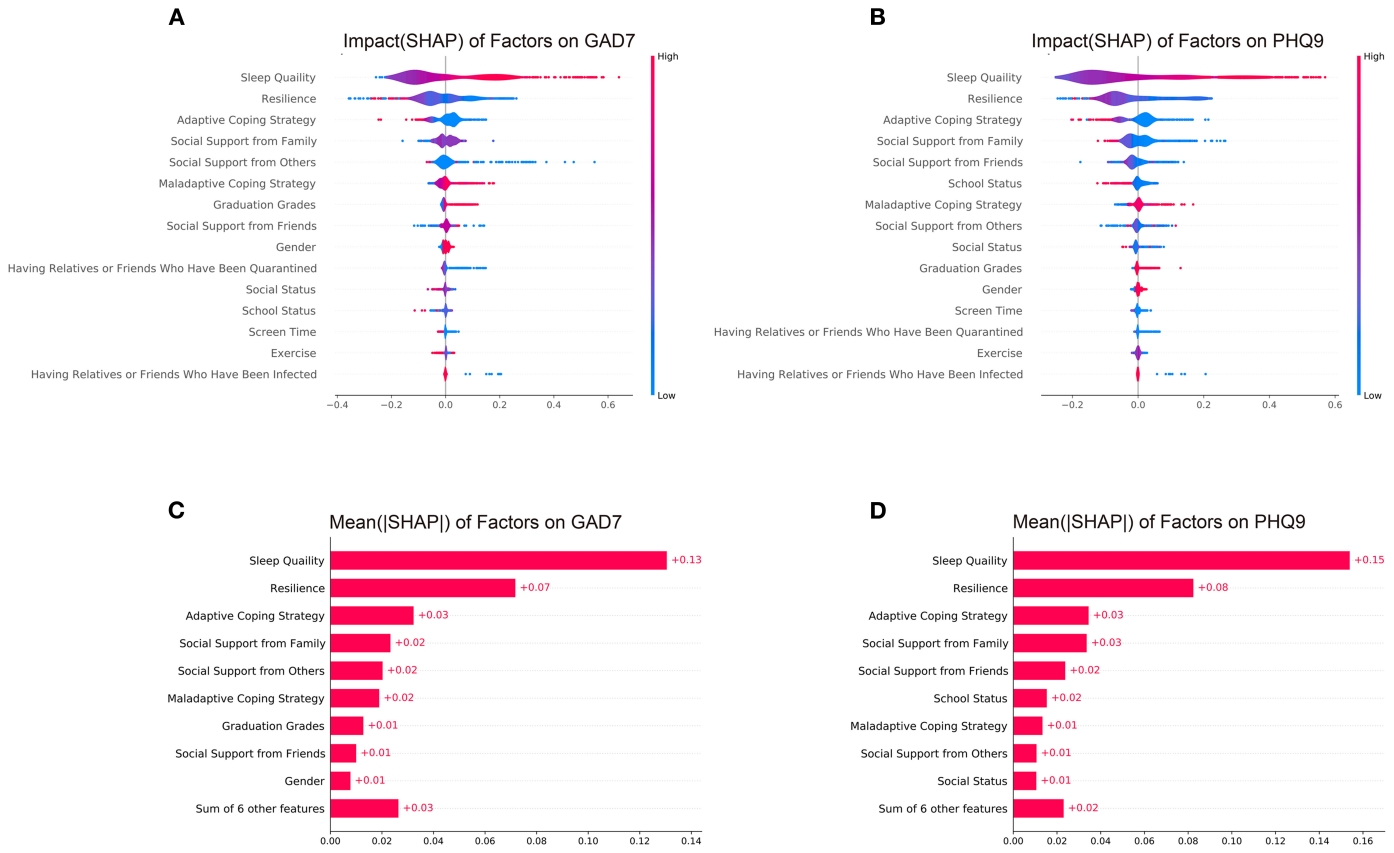
SHAP (Shapley additive explanations value) of all features. **(A)** SHAP of factors for GAD-7; **(B)** SHAPs of factors for PHQ-9; **(C)** mean (|SHAP|) of factors for GAD-7; **(D)** mean (|SHAP|) of factors for PHQ-9. |SHAP| represents the absolute value of SHAP.

As shown in Figure 1 and Table 2, we found that the GBDT+LR model had the best performance for both the GAD-7 (average AUC = 0.819) and PHQ-9 (average AUC = 0.857). Because GBDT is a boosted decision tree model, it was difficult to accurately assess the impact of each feature on the dependent variables. We applied the SHAP package to calculate the SHAP, which measures how features affect the dependent variables of every factor. To be more specific, the SHAP is able to explain the results of machine learning models based on game theory (51). Figures 2A,B show the distribution of each feature and the impact of each feature on the adolescents' mental health for two directions. We found that poor sleep quality, low psychological resilience, maladaptive coping strategy, low social support, being at graduation grades, being female, having relatives/friends who have been quarantined/infected, low perceived social status had negative impact on adolescents' mental health. We then calculated the mean of |SHAP| (absolute value of SHAP) to show the total impact of each feature (or feature importance) on the mental health of adolescents (no matter whether the directions were the same or the opposite). As shown in Figures 2C,D), the mean (|SHAP|) ranking list was sleep quality, resilience, adaptive coping strategies, social support from family, social support from others, maladaptive coping strategies, graduation grades, social support from friends, gender, and others for anxiety. Similarly, the mean (|SHAP|) ranking list was sleep quality, resilience, adaptive coping strategies, social support from family, social support from friends, school status, maladaptive coping strategies, social status, social support from others, and others for depression. Based on these two lists of feature importance ranking, we proposed a five-step mental health education strategy to be used during the COVID-19 pandemic (sleep quality-resilience-coping strategies-social support-perceived social status).

**Table 2:** Evaluation of the four machine learning algorithms for anxiety and depression.

**Models**	**AUC (95% CI)**	**Sensitivity (95% CI)**	**Specificity (95% CI)**
**Anxiety**			
LR	0.80 (0.75, 0.86)	0.72 (0.66, 0.78)	0.72 (0.67, 0.78)
KNN	0.80 (0.76, 0.86)	0.73 (0.67, 0.79)	0.73 (0.67, 0.79)
GBDT	0.81 (0.77, 0.86)	0.73 (0.69, 0.78)	0.73 (0.69, 0.78)
GBDT+LR	0.82 (0.76, 0.88)	0.74 (0.68, 0.80)	0.74 (0.68, 0.80)
**Depression**			
LR	0.85 (0.81, 0.90)	0.77 (0.72, 0.83)	0.77 (0.72, 0.83)
KNN	0.85 (0.80, 0.89)	0.75 (0.70, 0.81)	0.75 (0.70, 0.81)
GBDT	0.86 (0.82, 0.89)	0.77 (0.72, 0.81)	0.77 (0.73, 0.82)
GBDT+LR	0.86 (0.82, 0.89)	0.77 (0.72, 0.82)	0.77 (0.72, 0.82)

AUC, area under curve; KNN (k-nearest neighbors); LR (logistic regression); GBDT (gradient-boosted decision tree); GBDT+LR (a combination of GBDT and LR); CI, confidence interval. 95% CI = (mean−1.96^*^ standard deviation, mean+1.96^*^ standard deviation).

Conventionally, logistic regression models have been used in psychological and other medical areas to measure the influence of every feature more precisely by calculating the odds ratio (OR). Because the LR model also performed well on our datasets, here, we present the results from the LR model (Table 3).

**Table 3:** The results of the logistic regression model for anxiety and depression.

**Factors**	**Anxiety**		**Depression**	
	**OR (95% CI)**	***P-value***	**OR (95% CI)**	***P-value***
Gender (reference: male)	1.38 (1.08,1.77)	0.011^*^	1.29 (0.99,1.67)	0.055
Graduation grade (reference: no)	1.76 (1.24,2.50)	0.002^**^	1.58 (1.09,2.29)	0.017^*^
Having relatives or friends	0.16 (0.02,1.32)	0.088	0.03 (0.00,0.47)	0.012^*^
Who had been infected (reference: no)				
Having relatives or friends	0.49 (0.32,0.76)	0.001^**^	0.63 (0.39,0.99)	0.047^*^
Who had been quarantined (reference: no)				
Physical activity	0.93 (0.81,1.07)	0.283	1.00 (0.87,1.16)	0.972
Screen time	0.99 (0.89,1.10)	0.836	0.94 (0.85,1.05)	0.292
Sleep quality	1.18 (1.15,1.21)	0.000^***^	1.25 (1.22,1.29)	0.000^***^
Social status	1.01 (0.93,1.08)	0.889	0.98 (0.90,1.06)	0.585
School status	0.97 (0.90,1.05)	0.448	0.92 (0.85,0.99)	0.026^*^
Resilience	0.97 (0.95,0.99)	0.001^***^	0.97 (0.95,0.99)	0.000^***^
Social support from others	0.98 (0.93,1.03)	0.412	0.99 (0.94,1.05)	0.719
Social support from family	1.01 (0.97,1.05)	0.797	0.96 (0.92,1.00)	0.080
Social support from friends	1.01 (0.96,1.06)	0.695	1.00 (0.95,1.04)	0.867
Adaptive coping strategies	0.91 (0.87,0.96)	0.000^***^	0.93 (0.88,0.97)	0.001^**^
Maladaptive coping strategies	1.10 (1.04,1.16)	0.001^***^	1.10 (1.04,1.17)	0.001^***^

*OR, odds ratio;*

* *P < 0.05;*

** *P < 0.01;*

*** P < 0.001; CI, confidence interval. 95% CI = (mean−1.96* standard deviation, mean+1.96* standard deviation).

## Discussion

In this cross-sectional study, we investigated the current mental status of Chinese adolescents. The rates of occurrence of anxiety and depression symptoms were 28.3 and 30.8%. Compared with the rates at the beginning of the outbreak (13), adolescents' mental status has improved, but the outlook is still not optimistic. Wang et al. performed a meta-analysis in 2018, collecting the adolescents' depression/anxiety data in China before the COVID-19. The pooled occurrence rate of depression is around 21.73% in overweight subjects and 17.96% in non-overweight subjects; the pooled occurrence rate of anxiety is around 39.80% in over-weight subjects and 13.99% in non-overweight subjects (52). The occurrence rates were lower than 30.8% for depression and 28.3% for anxiety that we investigated in this study. Then, we used machine learning measures to screen for the factors that were most relevant to mood disorders among Chinese adolescents during the COVID-19 pandemic and utilized the SHAP to interpret our GBDT (anxiety) and GBDT+LR (depression) models to show the impact of each psychological factor.

Sleep quality had the closest relation with mood disorders among Chinese adolescents. Traditionally, psychiatrists/psychologists have treated insomnia and other sleep disorders as secondary symptoms that are mainly caused by psychiatric disorders, which account for >50% of insomnia cases. However, studies in adolescents indicate that sleep problems could directly contribute to subjective sleepiness (53), cognitive impairment (54), and mood disorders (55). Lo et al. performed a random controlled trial in 56 healthy adolescents and demonstrated that 1-week sleep restriction impaired adolescents' mood, and they did not fully recover even after two nights of sleep recovery (56). Therefore, sleep disorders and mood disorders affect each other: sleep disorders can accompany anxiety/depression, and anxiety/depression can further aggravate sleep disorders. In highly competitive societies, such as in China and other East Asian countries, adolescents (especially junior/senior high school students) face enormous academic pressure in the entrance examinations. They often voluntarily or are forced to spend more time studying at the expense of sleep. A very recent meta-analysis reported that the overall prevalence of sleep disturbance among Chinese adolescents was 26%, which was higher than that among university students (25.7%), and adults (20.4%) (57). Schools' student support services should take measures to ensure that students have adequate time for sleep and to enhance their sleep quality, including by reducing schoolwork stress and improving the living conditions in boarding schools. Furthermore, schools should find out if students have sleep disorders and provide psychological assistance in time.

The WHO reported that 50% of all psychological problems start by the age of fourteen, but most cases are undetected and untreated (58), and the outbreak of COVID-19 raised the risk of psychiatric disorders in adolescents. Early mental health education can prevent adolescents from having severe psychiatric outcomes. Practical mental health education implies that the psychological interventions are provided for all students without screening in order help students avoid risk factors and to enhance the protective factors. However, practical education strategies are limited. Based on the feature importance (SHAP) of the psychological factors, we proposed a five-step practical mental health education strategy for use during the COVID-19 pandemic. The first step is to emphasize the significance of healthy sleep and to teach students how to get good sleep and deal with sleep disorders (sleep quality). The second step is to foster adolescents' ability to bounce back with new learning and strength when facing adversity (resilience). The third step is to share with students strategies for the management of uncomfortable emotions by using healthy coping skills and avoiding unhealthy coping skills (coping strategies). The fourth step is to encourage students to build authentic relationships with their families, friends, teachers, and others (social support). The last step is to help students discover their core values and encourage students to ask for help when they encounter unfair treatment (social/school status). In practice, it is unnecessary to implement all five steps for all adolescents. Schools can set up online courses to complete the first, second, and third steps. For students who show symptoms of early mood disorders, school psychologists should provide them with accurate face-to-face psychological counseling and should finish the five steps. Besides, a very recent study demonstrated that expanding the testing capacity and applying strict control measures can effectively decease the total number of COVID-19 cases. Therefore, we highly recommend that schools could perform routine COVID-19 test for all the teachers and students (59).

We also found that some demographic characteristics and personal living styles were relevant to mood disorders. Adolescents who were female, were in graduation grades, and had relatives or friends who had been infected by COVID-19 or quarantined were more likely to develop mood disorders. In addition, adolescents who held relatively low subjective social and school status were susceptible to mood disorders. School psychologists should pay more attention to the mental health of students with these specific characteristics. Furthermore, schools can never discriminate against students due to their academic performance, family socioeconomic status, or gender, and they must propose a comprehensive and robust anti-bullying policy in order to protect students from bullying at school. Every adolescent should be free from discrimination or degrading treatment in school. As for personal living styles, we found that more physical activity and shorter screen time were related to healthier mental status. A wide range of investigations have shown that physical activity is beneficial for mental health in adolescents. Biddle et al. proved that there is a causal association between physical activity and cognitive functioning and depression (60). Currently, adolescents and children experience electronic devices as an indispensable part of their lives. Recent studies have linked screen time to mood disorders and even suicide among adolescents (61, 62), which could be mediated by inadequate sleep (63). Therefore, schools should encourage students to balance screen time and physical activity with other proper development and wellbeing activities.

This study has some strengths. First, the machine learning algorithms were reliable and appropriate. The AUCs were 0.819 for anxiety and 0.857 for depression, indicating that the factors that we screened had a close relationship with mood disorders in adolescents. Second, we collected the subjects without selection bias. The participants were from three independent junior high schools, three independent senior high schools, and three independent colleges (considering that the academic level of the school might influence students' mental status, we selected the schools of different levels on a 1:1:1 scale).

This study still has some limitations. Firstly, we did not explore the causal relationship between anxiety/depression and the screened factors because our statistical results are based on cross-sectional data, but not cohort data. A prospective cohort study is still needed. Secondly, our study was performed in Shandong province, China. The clinical value of our conclusions may be limited in other areas. Thirdly, this investigation was performed during Apr.20–Apr.30, 2021, when the COVID-19 in China had been well-controlled. Therefore, the results may have bias. Fourthly, due to ethic requirements, children (aged under 12) and adolescents who have recently suffered severe negative life events or were diagnosed with severe psychological disorders were not invited, which may also lead to selection bias. Finally, we did not perform a pre-survey to select included features. There might exist other significant psychological factors influencing adolescents' mental health. Besides, all the psychological scales included in this study were self-reported. This may lead to systematic measurement errors that either inflate or deflate the observed relationships between constructs, generating both Type I and Type II errors.

## Data Availability Statement

The original contributions presented in the study are included in the article/Supplementary Materials, further inquiries can be directed to the corresponding author/s.

## Ethics Statement

The studies involving human participants were reviewed and approved by The Ethics Committee of Shandong University. Written informed consent from the participants' legal guardian/next of kin was not required to participate in this study in accordance with the national legislation and the institutional requirements.

## Author Contributions

ZR and ZW Data collection was performed. YX Statistical analyses were performed. ZR and YX The manuscript was written. DL, YX, ZR, and ZW The study was designed and Contributed equally to this research. DL, RH, and CH The manuscript was revised. All authors read and approved the final manuscript.

## Conflict of Interest

The authors declare that the research was conducted in the absence of any commercial or financial relationships that could be construed as potential conflicts of interest.

## Publisher's Note

All claims expressed in this article are solely those of the authors and do not necessarily represent those of their affiliated organizations, or those of the publisher, the editors and the reviewers. Any product that may be evaluated in this article, or claim that may be made by its manufacturer, is not guaranteed or endorsed by the publisher.
